# *Paliurus spina-christi* Mill fruit extracts improve glucose uptake and activate the insulin signaling pathways in HepG2 insulin-resistant cells

**DOI:** 10.1186/s12906-023-03977-y

**Published:** 2023-05-08

**Authors:** Seyedeh Mona Mousavi Esfahani, Parastoo Tarighi, Kosar Dianat, Tabarek Mahdi Ashour, Negar Mottaghi-Dastjerdi, Mehdi Aghsami, Mahsa Sabernavaei, Hamed Montazeri

**Affiliations:** 1 Department of Medical Biotechnology, Faculty of Allied Medical Sciences, Iran University of Medical Sciences, Tehran, Iran; 2 Department of Pharmacognosy and Pharmaceutical Biotechnology, School of Pharmacy, Iran University of Medical Sciences, Tehran, Iran; 3 Department of Pharmacology and Toxicology, School of Pharmacy, Iran University of Medical Sciences, Tehran, Iran

**Keywords:** *Paliurus spina-christi* Mill, Diabetes, AMP-activated protein kinase (AMPK), AKT, GLUT1, GLUT4, Insulin receptor (INSR)

## Abstract

**Background:**

*Paliurus spina-christi* Mill. (PSC) fruit is frequently used in the treatment of diabetes mellitus in Mediterranean regions. Here, we investigated the effects of various PSC fruit extracts (PSC-FEs) on glucose consumption and some key mediators of insulin signaling pathways in high glucose and high insulin-induced insulin-resistant HepG2 cells.

**Methods:**

The effects of methanolic, chloroform and total extracts on cell proliferation were assessed by the MTT assay. The potential of non-toxic extracts on glucose utilization in insulin-resistant HepG2 cells was checked using a glucose oxidase assay. AKT and AMP-activated protein kinase (AMPK) pathway activation and mRNA expression levels of insulin receptor (INSR), glucose transporter 1 (GLUT1), and glucose transporters 4 (GLUT4) were determined by western blotting and real-time PCR, respectively.

**Results:**

We found that high concentrations of methanolic and both low and high concentrations of total extracts were able to enhance glucose uptake in an insulin-resistant cell line model. Moreover, AKT and AMPK phosphorylation were significantly increased by the high strength of methanolic extract, while total extract raised AMPK activation at low and high concentrations. Also, GLUT 1, GLUT 4, and INSR were elevated by both methanolic and total extracts.

**Conclusions:**

Ultimately, our results shed new light on methanolic and total PSC-FEs as sources of potential anti-diabetic medications, restoring glucose consumption and uptake in insulin-resistant HepG2 cells. These could be at least in part due to re-activating AKT and AMPK signaling pathways and also increased expression of INSR, GLUT1, and GLUT4. Overall, active constituents present in methanolic and total extracts of PCS are appropriate anti-diabetic agents and explain the use of these PSC fruits in traditional medicine for the treatment of diabetes.

**Supplementary Information:**

The online version contains supplementary material available at 10.1186/s12906-023-03977-y.

## Background

Type 2 diabetes mellitus (T2DM) is a biochemical and hormonal disease that already causing a global crisis. It is defined by increased glycemia caused by insulin-resistance and β-cell dysfunction, along with related insulin insufficiency [1]. Currently, T2DM accounts for about 90% of diabetes cases, and its incidence has substantially grown more than anticipated, especially in young individuals [2, 3]. Moreover, T2DM contributes to various clinical consequences, including cardiovascular diseases and renal insufficiencies, which adversely affect the quality of life through societal expenses [3].

Numerous medications are synthesized and available in the worldwide market as standard diabetes treatment. However; considering these drugs as a life-time program, adverse reactions may limit their use and patient compliance. In this regard, medicinal plants which have been used since time immemorial are of great interest in finding some cost-effective alternative or adjuvant therapy with fewer side effects. *Paliurus spina-christi* Mill. is a wild-standing shrub with greenish-yellow flowers commonly used as an antidiabetic remedy in the Middle East and Mediterranean region. *Paliurus spina-christi* Mill. which is a medicinal plant of genus Paliurus and family Rhamnaceae, is mainly determined in sunny and forest areas in the different provinces of Iran, such as Gorgan, Mazandaran, Hamedan, East, and West Azerbaijan, Zanjan, and Northeast Khorasan. This plant has five different species, and its therapeutic potential can be attributed to large amounts of alkaloids, flavonoids, polyphenols, and tannins [4, 5]. In recent studies, the biological activity of PSC active constituents, such as quercetin, catechin, rutin, etc., have been shown to have a significant therapeutic impact on diabetes brought on by oxidative stress [6]. Also, the positive effects of PSC-FEs on the catalase and superoxide dismutase enzyme expression as well as inhibition of lipid peroxidation were proved in streptozotocin-induced diabetic rats [4]. According to another study, the ethanol and ethyl acetate extracts of PSC branches and leaves considerably reduced inflammation through their antioxidant activity [7]. Among the proposed medical properties of this plant, anti-hypercholesterolemic, anti-diarrheal, anti-rheumatic, and diuretic effects can be mentioned [8]. Moreover, *Paliurus spina-christi* Mill. fruit has been reported to have anti-inflammatory, antioxidant, and antidiabetic activities [4–6], though the exact mechanisms are yet to be identified.

AMP-activated protein kinase (AMPK) is a signaling pathway implicated in T2DM which may accelerate ATP production via glycolysis and β–oxidation [9], and activation of AMPK is associated with enhancement of glucose transporter 1 (GLUT1) [10]. Also, AMPK can stimulate the hepatic signaling pathway phosphatidylinositol 3-kinase (PI3K) /AKT, which is essential for insulin resistance and glucose metabolism regulation, as it’s one of several effectors associated with T2DM [11, 12]. Furthermore, PI3K signaling is required for the glucose transporter 4 (GLUT4) transport pathway [13].

While previous studies have shown the efficacy of *Paliurus spina-christi* Mill. fruits on diabetes, no research has been done to directly interrogate the antidiabetic effect of PSC fruit extracts (PSC-FEs) on glucose consumption and principal mediators of the insulin signaling pathway in insulin-resistant HepG2 cells. Furthermore, flavonoids include compounds such as epigallocatechol, quercetin, rutin, catechin, phenolic acids, etc. that have significant antidiabetic potential because of their immunoregulatory regulation of blood sugar transporters, which includes boosting GLUT4 translocation via PI3K/AKT and AMPK pathways [14]. Therefore, in this study, HepG2 insulin-resistant cells were established to figure out the consequences of PSC-FEs on AKT and AMKP activation, and also the expression of insulin receptor (INSR) and major glucose transporters involved in glucose metabolism. These findings could enhance our understanding of anti-hyperglycemic mechanisms and the usefulness of PSC-FEs in T2DM.

## Materials and methods

### Preparation of the PSC fruit extracts

The *Paliurus spina christi* Mill. plant was collected by authors from the Hamedan province (Kaboodar ahang). Botanist Prof. Gholamreza Amin deposited taxonomic identification with the code 7084-TEH in the Faculty of Pharmacy, Tehran University of Medical Sciences which is available mainly to researchers. The Ethics Committee of Iran University of Medical Sciences approved the study protocol under the code IR.IUMS.REC.1399.1266, Tehran, Iran. The experiments were performed at the School of Pharmacy, Iran University of Medical Sciences (Tehran, Iran, 2019). Freshly dried and healthy plant material was ground into fine powder in an electric grinder. To prepare the total extract, 250 g of the air-dried aerial parts of the plant were macerated with 70% methanol at room temperature. The crude extract (90 g) was fractionated via liquid-liquid extraction with petroleum ether, chloroform, ethyl acetate, and methanol respectively. The extracts were solubilized in DMSO not exceeding 1% and kept at -20 ℃ till conducting the assays.

### Phytochemical tests on PSC total extract [15, 16]

Test for alkaloids.

The total extract (2 g) was heated and purified with almost 1 ml of HCl. Mayer reagent was used to treat 2 ml of supernatant independently. The presence of alkaloids was discovered in turbidity or precipitation, with an orange color.

Test for flavonoids.

One method of identifying flavonoids is the cyanidin test. In this experiment, flavonoids produce a pink-to-red color in an acidic environment in existence of magnesium chips.

Test for Saponins.

Total extract (1 g) was added to 2 ml of heated water in a petri dish, allowed to cool and properly mixed. Saponins can be detected by the formation of foam.

Test for Steroids.

2 ml of chloroform and 1 ml of sulfuric acid (H2SO4) were added to 1 ml of total extract. The existence of steroids is shown by the appearance of a reddish-brown ring at the contact.

Test for tannins.

In a petri dish, approximately 1 g of total extract was heated in 20 ml of distilled water and then filtered. 1 ml of the extract was added with 5% FeCl3 (1 ml) and 1% gelatin solution. The existence of tannins was shown by the development of green color.

Test for cyanogenic glycosides.

About 2 g of total extract with adequate water was mixed in a test tube, and then the sodium-impregnated paper was added. The test result is positive when the color of the paper is changed from yellow to red.

### Cell culture and induction of insulin-resistant model

The HepG2 cell line was purchased from the Iranian Biological Resource Center (IBRC). The cell line was cultivated in DMEM-high glucose (25mM glucose) medium (Biowest, Nuaille, France) enriched with 10% Fetal Bovine Serum (Gibco) and 1% penicillin/streptomycin (Biowest, Nuaille, France) at 37 °C in a 5% CO2 incubator.

Insulin-resistant HepG2 cell model was induced according to a previously described method [17]. After plating in a 24-well plate and overnight attachment, the cells were incubated in serum-free DMEM for 6 h. Subsequently, the cells were treated with 1 μm of regular insulin (Ronak Pharmaceuticals, Iran) and the induction was carried out for 24 h.

### Cell viability assay

The MTT assay was used to assess the impact of PSC-FEs on cell viability. HepG2 cells were implanted at a density of 105 cells per well in 96-well plates. 24 h later, the cells were treated with different doses of total, methanolic, and chloroform extracts of PSC, ranging from 500 to 5 ug/ml. The day after, the next day, the cells were rinsed with PBS. The cells were then treated with 20ul of 5 mg/ml of MTT (Sigma, Germany) solution and incubated in dark for 4 h. The resultant formazan was solubilized by adding 60 ul of DMSO (Sigma, Germany) and the optical density was quantified spectrophotometrically with a BioTek microplate reader at 570 nm (reference filter, 690 nm). MTT assay was also performed in GOP-POD enzyme assay to normalize cell number to glucose consumption.

### Glucose oxidase-peroxide (GOD-POD) enzyme assay

The GOD-POD assay was carried out to determine the glucose levels using Glucose Assay Kit (Pars Azmun, Iran) by the manufacturer’s protocol. In the first, cells were seeded into a 12-well plate at a density of 10^5^ cells per well. 24 h after induction of insulin resistance as described above, the cells were treated with either metformin or different concentrations of PSC-FEs dissolved in phenol red-free DMEM-high glucose. 24 h following treatment, 10 ul of the culture supernatants were collected and used for the GOD-POD assay.

### RNA isolation and quantitative RT-PCR

HepG2 cells were seeded in 6-well plates and RNA extraction was done following induction of insulin resistance and treatment with either one of the two 50ug/ml of PSC-FEs or 1mM of metformin, using Tripure isolation reagent (Roche, Switzerland). The amount and purity of the RNA were evaluated by recording the absorbance at 260 and 280 nm. Total RNA was reverse transcribed into cDNA using easy cDNA synthesis kit (Parstous, Iran) in accordance with manufacturer’s manual. The quantitative real-time PCR and gene-specific primers (Table 1) were performed on a LightCycler® instrument using the SYBR Premix Ex Taq II (Takara, Japan). The amplification program included an initial denaturation step at 95 °C for 5 min, followed by 40 cycles of denaturation at 95 °C for 5 s, annealing, and extension at 60 °C for 30 s, and a final extension of 10 min at 72 °C. To calculate the relative gene expression, the expression of target genes was normalized to Glyceraldehyde 3-phosphate dehydrogenase (GAPDH) expression, and the fold change was determined according to the 2^−ΔΔCt^ method.

**Table 1:** GLUT1, GLUT9, INSR and GAPDH primer sequences, product length and annealing temperatures. bp, base pair; FW, forward primer; RV, reverse primer

Gene		Sequence	Product bp	Annealing temperature
GLUT1	FW RV	TCTGGCATCAACGCTGTCTTC CGATACCGGAGCCAATGGT	94	60
GLUT4	FW RV	AGTCTTCACCTTGGTCTCGG CAGAGCCACAGTCATCAGGA	112	60
INSR	FW RV	AAAACGAGGCCCGAAGATTT GAGCCCATAGACCCGGAAG	90	60
GAPDH	FW RV	GAAGGTGAAGGTCGGAGTCAAC CAGAGTTAAAAGCAGCCCTGGT	71	60

### Preparation of cell lysates and western blot analysis

Western blotting was used to investigate the effects of PSC-FEs on the AMPK/AKT signaling pathways. Insulin-resistant HepG2 cells in 6-well plates were treated for 24 h with high and low concentrations (5 and 50 ug/ml) of total and methanolic PSC-FE and also metformin (1 mM) as the positive control. The treated and untreated cells were washed with ice-cold PBS and lysed with RIPA buffer supplemented with protease tablets and phosphatase inhibitor cocktail tablets on ice for 30 min. These cell lysates were centrifugated for 20 min at 25,000 g at 4 °C to remove the insoluble material. Protein concentration was determined by the BCA protein assay kit according to the corresponding user manual. Protein samples were separated by 10% SDS-polyacrylamide gel electrophoresis and transferred to PVDF (Roche Diagnostics GmbH, Germany) membranes. After blocking with 5% BSA in tris-buffered saline containing 0.1% Tween 20 (TBST) for 1 h at room temperature, the blots were probed overnight at 4°c with primary antibodies. The membranes were then washed three times with TBST for 10 min each and incubated with appropriate secondary antibodies for 1 h. Subsequently, the blots were washed three times with TBS and the luminescence was detected using BM Chemiluminescence Western Blotting Kit (Roche, Switzerland). In all experiments, β-actin was used as loading control and the intensities were quantified by ImageJ software (NIH, USA).

### Statistical analysis

Multiple comparisons between data sets were carried out using one-way analysis of variance (ANOVA) followed by *Tukey’s post hoc* test, with a significance of *p* < 0.05 for comparing treated and untreated samples. The data is shown as mean ± SD (standard deviation) of three independent experiments.

## Results

### Phytochemical analysis of PSC total extract

According to the results of phytochemical tests listed in Table 2, the total extract of PSC does not contain any saponins or cyanogenic glycosides, but it contains high amount of alkaloids, tannins, steroids, and flavonoids. According to the previous research results on PSC’s composition, such findings were not unexpected [4].

**Table 2:** Phytochemical analysis of total extract of PSC fruits

Type of examination	Unit	Result
Alkaloids	Sedimentation in the presence of Mayer reagent	+
Flavonoids	No red color in cyanidin test	+
Saponin	Lack of stable foam	-
Steroids	Creates a red color after adding Sulfuric acid	+
Tannins	Sedimentation with ethyl acetate solution	+
Cyanogenic glycosides	Do not change the color of the paper from yellow to red	-

### Cell viability and proliferation

**Fig. 1:**
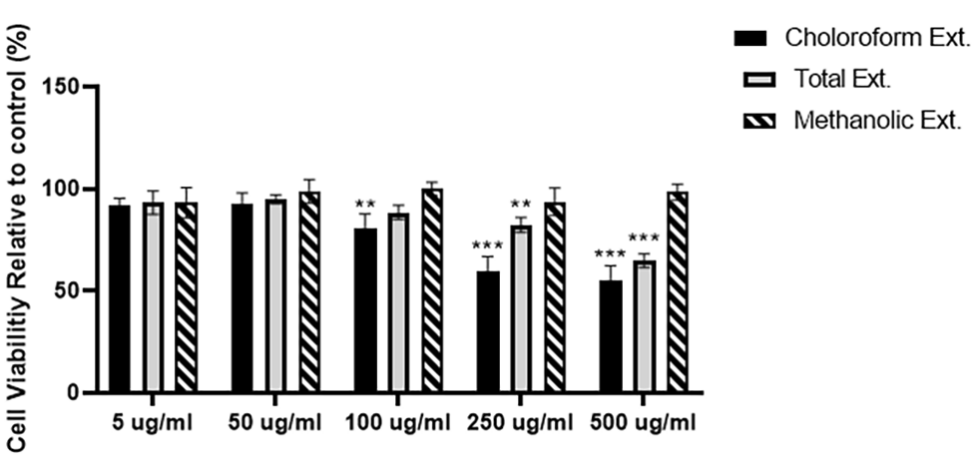
Cytotoxic effects of different concentrations of PSC total, methanolic and chloroform fruit extracts on cell viability in HepG2 liver cells. Cells were treated with different concentrations (5, 50, 100, 250 and 500 ug/ml) of PSC-FEs for 24 h and cell viability was determined and expressed as a percentage relative to the vehicle group. Values are mean ± SD from three individual experiments performed in octuplicate. *p < 0.05, ** p < 0.01, and *** p < 0.001 compared with untreated HepG-2 cells (control)

A human hepatic cell line was used to assess the possible cytotoxicity effects of several compounds (HepG2 cells). The cells were exposed to different concentrations (5, 50, 100, 250, and 500 µg/ml) of total, methanolic, and chloroform extracts of PSC fruits for 24 h. As determined by the MTT assay, among the three investigated extracts, the chloroform-based one showed the highest toxicity, with an IC50 value of around 500 ug/ml. However, methanolic extract showed no significant toxicity at concentrations of 1000 ug/ml or lower, and interestingly, cell growth inhibition was not observed in the range of total extract concentrations used in this study (Fig. 1). As a result of the low/zero toxicity of methanolic and total extracts, concentrations of 5(low) and 50 (high) ug/ml of these two extracts were chosen for further investigations.

### Effect of PSC-FEs on glucose consumption in insulin-resistant HepG2 cells

**Fig. 2:**
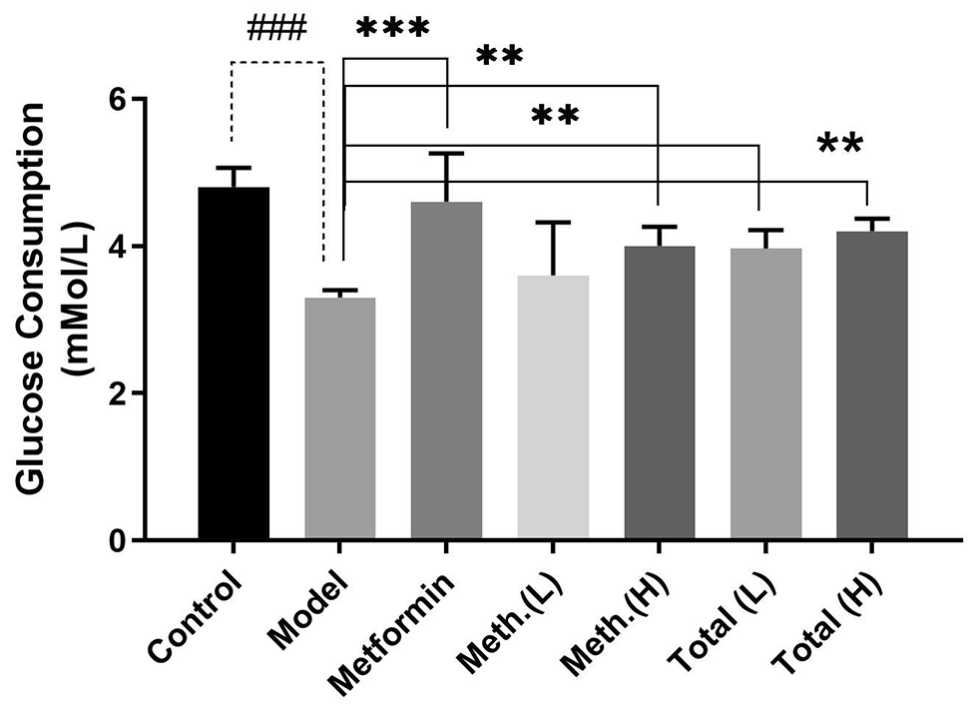
Effects of PSC-FEs on glucose consumption in insulin-resistant HepG2 cells. The GOD-POD assay was carried to check glucose consumption. The insulin-resistant HepG2 cells were treated with low (5 ug/ml) and high (50 ug/ml) doses of total and methanolic PSC-FE for 24 h and glucose consumption was measured as described under materials and methods. Treatment with 1 mM metformin was considered as positive control. Values are mean ± SD from three individual experiments performed in triplicate. *p < 0.05, ** *p* < 0.01, and *** *p* < 0.001 compared with untreated insulin-resistant cells (model). ### *p* < 0.001 compared with untreated HepG2 cells (control)

As shown in Fig. 2, glucose consumption was dramatically reduced in insulin-resistant cells compared to control cells (*p* < 0.001). Besides, this induction exhibited no cytotoxicity, indicating that a model of insulin resistance in a hepatic cell line was successfully generated.

The positive control, metformin, showed the foremost restorative effect on glucose consumption in insulin-resistant cells (*p* < 0.001). Similarly, total extract of PSC-F enhanced glucose consumption at both low and high concentrations (*p* < 0.01). The ability of methanolic extract was evident only at high concentrations, though (*p* < 0.01).

### Effects of PSC-FEs on phosphorylated and total levels of AMPK in insulin-resistant HepG2 cells

**Fig. 3:**
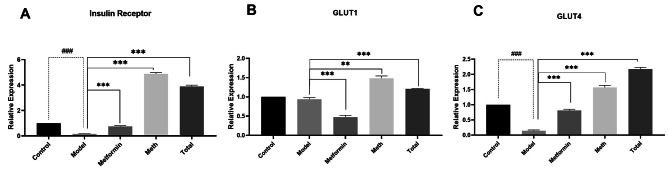
Effects of PSC-FEs on the expression of (**A**) insulin receptor, (**B**) GLUT1 and (**C**) GLUT4 in insulin-resistant HepG2 cells. The insulin-resistant HepG2 cells were treated with 50 ug/ml of total and methanolic PSC-FEs and used for RNA extraction 24 h after treatment. Total RNA was reverse transcribed and used a template in quantitative real-time PCR. Treatment with 1 mM metformin was considered as positive control. Data represent mean ± SD from three individual experiments performed in triplicate. ** *p* < 0.01 and *** *p* < 0.001 compared with untreated insulin-resistant cells (model). ## *p* < 0.01 and ### *p* < 0.001 compared with untreated HepG2 cells (control)

To investigate the effects of PSC-FEs on AMPK activation, insulin-resistant HepG2 cells were incubated with high and low concentrations of total and methanolic extracts, as well as metformin as a positive control, for 24 h, and cell lysates were evaluated for total and phosphorylated protein by western blotting (Fig. 3a). As depicted in Fig. 3b, insulin resistance induction drastically decreased p-AMPK and AMPK. This reduction was reverted upon metformin treatment and also at both concentrations of total extract (*p* < 0.001). However, reversion by methanolic extract was seen only at high concentration (*p* < 0.01).

### Effects of PSC-FEs on phosphorylated and total levels of AKT in insulin-resistant HepG2 cells

To check the modulation of AKT by PSC-FEs, insulin-resistant HepG2 cells were incubated with high concentrations of total and methanolic extracts. The total and phosphorylated AKT were assessed in cell lysates by western blotting (Fig. 3a). As illustrated in Fig. 3c, AKT activation was significantly reduced in insulin-resistant model. Incubation with metformin could improve degree of AKT activation (*p* < 0.01), however, similar effect was only seen at high concentrations of methanolic extract (*p* < 0.01) (Fig. 3c).

### Effects of PSC-FEs on the expression of INSR, GLUT1 and, GLUT4 in insulin-resistant Hep-G2 cells

**Fig. 4:**
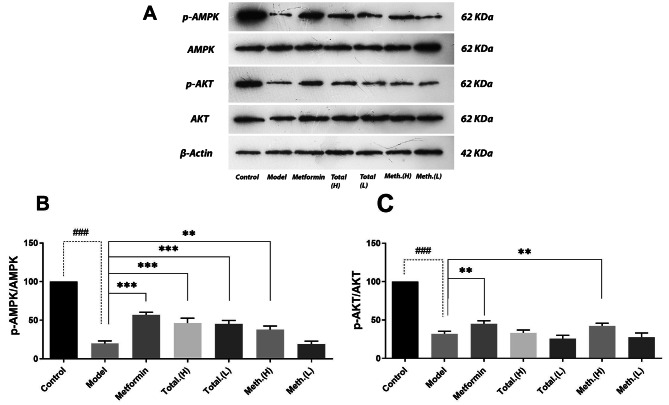
Effects of PSC-FEs treatment on AKT and AMPK phosphorylation in insulin-resistant HepG2 cells. After induction of insulin-resistance, HepG2 cells were treated with low (5 ug/ml) and high (50 ug/ml) doses of total and methanolic PSC-FE for 24 h. The cells were lysed and subjected to western blot analysis for AKT, p-AKT, AMPK and p-AMPK. Representative western blot for phosphorylated and total AKT and AMPK are shown in (**A**). Quantitative analysis of p-AKT/AKT and p-AMPK/AMPK are shown in (**B**) and (**C**), respectively. Treatment with 1 mM metformin was considered as positive control. Equal Protein loading was ensured by β-actin. Data represent mean ± SD from three individual experiments performed in triplicate. ** *p* < 0.01 and *** *p* < 0.001 compared with untreated insulin-resistant cells (model). ### *p* < 0.001 compared with untreated HepG2 cells (control)

In the subsequent attempt to define the mechanisms, we evaluated the changes in expression of three glucose metabolism-related genes. As shown in Fig. 4a, INSR expression diminished significantly upon induction of insulin resistance (*p* < 0.001). Although INSR expression was upregulated following treatment by metformin and both methanolic and total extracts compared to the untreated model (*p* < 0.001), interestingly, the amount of INSR expression in the extract-treated group seems to be much more than with metformin.

To better understand the effects of PSC-FEs, mRNA expression levels of GLUT1 and GLUT4 were also investigated. As shown in Fig. 4b, GLUT1 expression remained unchanged in insulin-resistant model but was significantly downregulated in metformin treated group. On the other hand, both applied extracts improved GLUT1 expression remarkably compared to the model group (*p* < 0.001). In terms of GLUT4 expression, we found its obvious downregulation following insulin resistance induction (*p* < 0.001) (Fig. 4c). Treatment with metformin, total and methanolic extracts, all recovered GLUt4 expression status by its upregulation (*p* < 0.001) (Fig. 4c), although this effect was more pronounced by extracts.

## Discussion

T2DM is among the most highly prevalent illnesses in the world. Although there are a large number of synthetic medicines such as metformin available on the market, adverse effects still influence patient compliance. Among the herbal medicines with far fewer side effects, PSC fruit has been used for treatment of T2DM in traditional medicine of the eastern Mediterranean region, and in a streptozotocin-induced diabetic group, its decoction showed antidiabetic action [18].

Recently, PSC-FEs has gained interest due to its antidiabetic effects. Two separate studies performed by Takim et al. demonstrated the positive effects of PSC-FEs on reducing the blood glucose level of streptozotocin-induced diabetic rats, mainly through its antioxidant activity and its potential to harness oxidative stress enzymes [4]. However, further molecular pathways behind PSC-FEs’ antidiabetic effects and potential toxicity have not yet been studied.

In this research, we looked into the impacts of PSC-FEs on two principal pathways involved in insulin resistance, i.e., AMPK and AKT [4, 19] and also on expression levels of INSR [20], GLUT1, and GLUT4 [21], as major regulators of glucose hemostasis.

HepG2 cells are a legitimate in vitro cell model of human hepatocytes in diabetes studies because of their preserved insulin downstream signaling pathways [22]. Verified by reduced glucose consumption, we used 25 mM glucose (DMEM, high glucose) plus 10^− 6^ M of insulin to develop an insulin-resistant HepG2 model. Treatment of insulin-resistant HepG2 cells with PSC-FEs caused an obvious increase in glucose consumption, indicating PSC-FEs can mitigate the induced insulin resistance in HepG2 cells.

To gain mechanistic insight on the increase in glucose consumption, the levels of activated AKT and AMPK were investigated. Treatment of HepG2 cells with high glucose and insulin for 24 h induced an intense impairment in AKT and AMPK degree of phosphorylation, whereas these inhibitions were restored by methanolic extract in the case of AKT and both total and methanolic extracts in the case of AMPK, explaining the post-treatment rise in glucose consumption. This improvement in p-AMPK/AMPK and p-AKT/AKT ratio, as pivotal effector molecules in glucose metabolism, can be explained by high flavonoid and other phenolic compounds present in PSC-FEs [4] including quercetin [23, 24], catechin [25], rutin [26], hesperidin [27, 28], kaempferol [29, 30], proanthocyanidin [31, 32], etc. as previously reported.

The downregulation of INR expression seen in the insulin-resistant model indicates attenuation of the insulin signaling pathway. This reduction was partially restored following metformin treatment, which is consistent with previous study in which the effect of insulin on human primary hepatocytes was examined [33]. Interestingly, both total and methanolic PSC-FEs drastically upregulated INR expression not only compared to the insulin-resistant model but also compared to the control group. Increased expression of INSR also has been reported in another study that showed that high flavonoid content herbal extracts were used to treat diabetic rats [34].

GLUT1 is known to be the major transporter of glucose in HepG2 cells located in plasma membrane [35]. Our results showed that metformin can lower expression of GLUT1, which is in line with the earlier study that metformin downregulated the level of GLUT1 in the liver cells of diabetic rats [36]. In contrast to metformin, methanolic and total extracts of PSC fruit enhanced expression of GLUT1, explained by their flavonoid concentration. Such enhancements were reported earlier upon administration of quercetin and rutin rich *Chimonanthus nitens* Oliv. leaf extract [37] and also following HepG2 treatment with quercetin and kaempferol [38]. Intriguingly, GLUT 1 expression is also a function of AMPK activation that is shown to be amplified in our investigation [39].

GLUT4 is a member of the PI3K/AKT signaling pathway that is translocated to the cellular membrane to mediate glucose inflow once the cascade is activated [40]. In this experiment, inducing insulin resistance diminished GLUT4 expression, indicating suppression of the PI3K/AKT pathway. Similar to other studies [41, 42] suppression was reverted incompletely by metformin treatment delineating various mechanisms of metformin for exerting its antidiabetic effects. The effects of methanolic and total extracts of PSC were even more pronounced in upregulation of GLUT4, as it far exceeded the level of expression in control cells. According to other investigations, catechin [43], epigallocatechin [44], hesperidin [45], kaempferol [46], quercetin [46], and rutin [47] present in PSC methanolic and total extracts have the potential to induce GLUT4 expression.

Altogether, these findings suggest that total and methanolic PSC-FEs could protect HepG2 cells from insulin resistance, as evidenced by enhancement of glucose uptake, enhancement of AMPK and AKT activation, and also an increase in expression of INSR, GLUT1, and GLUT4. Nevertheless, further investigations are required to validate PSC-FEs as natural antidiabetic agents.

## Conclusions

Ultimately, our results shed new light on methanolic and total PSC-FEs as sources of potential anti-diabetic medications, restoring glucose consumption and uptake in insulin-resistant HepG2 cells. These could be at least in part due to re-activating AKT and AMPK signaling pathways and also increased expression of INSR, GLUT1, and GLUT4. Overall, active constituents present in methanolic and total extracts of PCS are appropriate anti-diabetic agents and explain the use of these PSC fruits in traditional medicine for the treatment of diabetes.

## Electronic supplementary material

Below is the link to the electronic supplementary material.

**Additional file 1**

**Additional file 2**

**Additional file 3**

**Additional file 4**

**Additional file 5**

**Additional file 6**

**Additional file 7**

**Additional file 8**

**Additional file 9**

**Additional file 10**

**Additional file 11**

**Additional file 12**

**Additional file 13**

**Additional file 14**

## Data Availability

The datasets used and/or analyzed during the current study are available from the corresponding author on reasonable request.

## List of abbreviations

PSC-FEs: Paliurus spina christi fruit extracts
PSC: Paliurus spina-christi
T2DM: Type 2 diabetes mellitus
PI3K: Phosphatidylinositol 3-kinase
GAPDH: Glyceraldehyde 3-phosphate dehydrogenase
MAPK: Mitogen-activated protein kinases
INSR: Insulin receptor
GLUT1: Glucose transporters 1
GLUT4: Glucose transporters 4
