# Correction: *Paliurus spina-christi* Mill fruit extracts improve glucose uptake and activate the insulin signaling pathways in HepG2 insulin-resistant cells

**DOI:** 10.1186/s12906-024-04714-9

**Published:** 2024-11-26

**Authors:** Seyedeh Mona Mousavi Esfahani, Parastoo Tarighi, Kosar Dianat, Tabarek Mahdi Ashour, Negar Mottaghi-Dastjerdi, Mehdi Aghsami, Mahsa Sabernavaei, Hamed Montazeri

**Affiliations:** 1 Department of Medical Biotechnology, Faculty of Allied Medical Sciences, Iran University of Medical Sciences, Tehran, Iran; 2 Department of Pharmacognosy and Pharmaceutical Biotechnology, School of Pharmacy, University of Medical Sciences, Tehran, Iran; 3 Department of Pharmacology and Toxicology, School of Pharmacy, Iran University of Medical Sciences, Tehran, Iran

**Correction: BMC Complement Med Ther 23**,** 151 (2023)**

10.1186/s12906-023-03977-y

Following publication of the original article [1], the authors reported an error in Materials & Methods Section, page 2, right column, line 34. The identification code for *Paliurus spina christi* Mill. is given as 6761-TEH. The correct identification code is 7084-TEH.

The original article has been corrected.
