# Supplementary material for: Paliurus spina-christi Mill fruit extracts improve glucose uptake and activate the insulin signaling pathways in HepG2 insulin-resistant cells
Source: BMC Complement Med Ther. 2023 May 8;23:151. doi: 10.1186/s12906-023-03977-y (PMC10165757; doi:10.1186/s12906-023-03977-y)

Fluorescence Curves

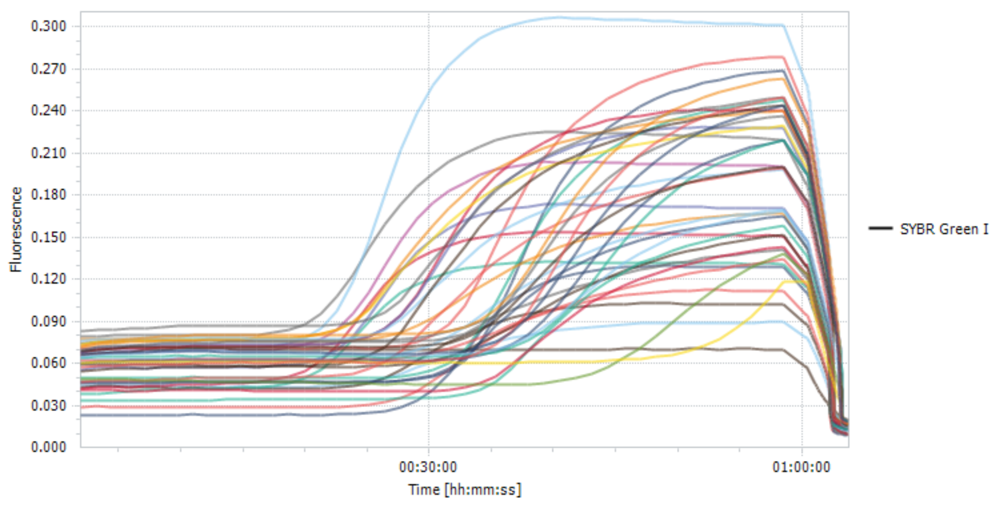

Integration Time

Program	Dye	Integration Time [ms]
2 Step Amplification	SYBR Green I	338
Melting	SYBR Green I	1000

Plate View

*	1	2	3	4	5	6	7	8	9	10	11	12
A												
B												
C												
D	U	U	U	U	U	U	U	U				
E	U	U	U	U	U	U	U	U				
F	U	U	U	U	U	U	U	U				
G	U	U	U	U	U	U	U	U				
H	U	U	U	U	U	U	U	U				

Sample Table

Color	Position	Sample Name	Gene Name	Condition Name	Sample Type	Standard	Dye
	D1	control-glut4	None		Unknown		SYBR Green I
	D2	control-glut4	None		Unknown		SYBR Green I
	D3	model-glut4	None		Unknown		SYBR Green I
	D4	model-glut4	None		Unknown		SYBR Green I
	D5	metaline-glut4	None		Unknown		SYBR Green I
	D6	metaline-glut4	None		Unknown		SYBR Green I
	D7	met-glut4	None		Unknown		SYBR Green I
	D8	met-glut4	None		Unknown		SYBR Green I
	E1	total-glut-4	None		Unknown		SYBR Green I
	E2	total-glut4	None		Unknown		SYBR Green I
	E3	control-glut1	None		Unknown		SYBR Green I
	E4	control-glut1	None		Unknown		SYBR Green I
	E5	model-glut1	None		Unknown		SYBR Green I
	E6	model-glut1	None		Unknown		SYBR Green I
	E7	metaline-glut1	None		Unknown		SYBR Green I
	E8	metaline-glut1	None		Unknown		SYBR Green I
	F1	met-glut1	None		Unknown		SYBR Green I
	F2	met-glut1	None		Unknown		SYBR Green I
	F3	total-glut1	None		Unknown		SYBR Green I
	F4	total-glut1	None		Unknown		SYBR Green I
	F5	control-Ins	None		Unknown		SYBR Green I
	F6	control-Ins	None		Unknown		SYBR Green I
	F7	model-Ins	None		Unknown		SYBR Green I
	F8	model-Ins	None		Unknown		SYBR Green I
	G1	metaline-Ins	None		Unknown		SYBR Green I
	G2	metaline-Ins	None		Unknown		SYBR Green I
	G3	met-Ins	None		Unknown		SYBR Green I

	G4	met-Ins	None		Unknown		SYBR Green I
	G5	total-Ins	None		Unknown		SYBR Green I
	G6	total-Ins	None		Unknown		SYBR Green I
	G7	control-gap	None		Unknown		SYBR Green I
	G8	control-gap	None		Unknown		SYBR Green I
	H1	model-gap	None		Unknown		SYBR Green I
	H2	model-gap	None		Unknown		SYBR Green I
	H3	metaline-gap	None		Unknown		SYBR Green I
	H4	metaline-gap	None		Unknown		SYBR Green I
	H5	met-gap	None		Unknown		SYBR Green I
	H6	met-gap	None		Unknown		SYBR Green I
	H7	total-gap	None		Unknown		SYBR Green I
	H8	total-gap	None		Unknown		SYBR Green I

Amplification Curves

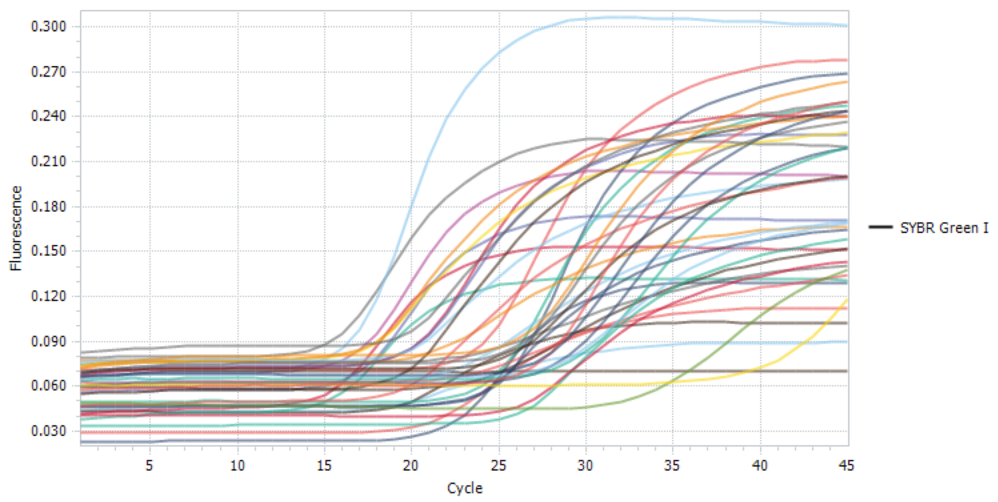

Melting Curves

Melting

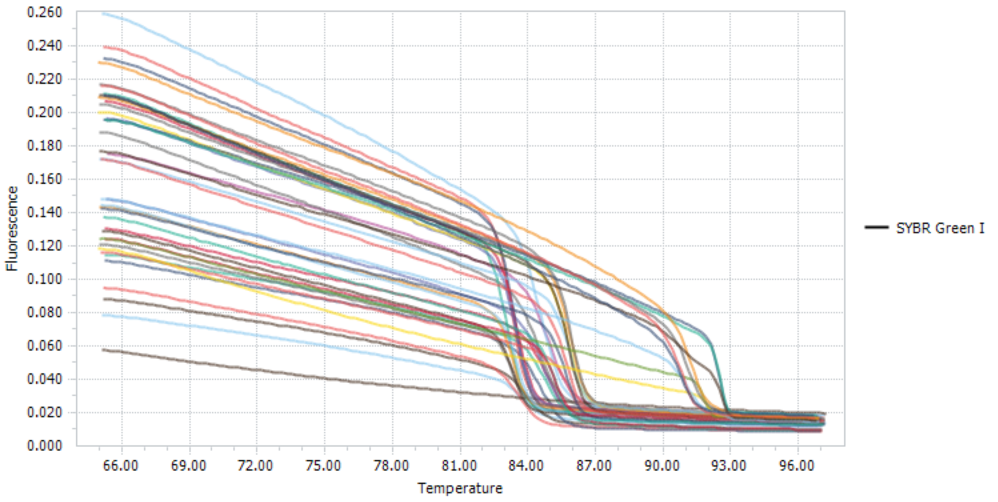

Supplement: Supplementary file 1 — Additional file 1 [file 12906_2023_3977_MOESM1_ESM.pdf]
