# Supplementary figures and images for: Paliurus spina-christi Mill fruit extracts improve glucose uptake and activate the insulin signaling pathways in HepG2 insulin-resistant cells
Source: BMC Complement Med Ther. 2023 May 8;23:151. doi: 10.1186/s12906-023-03977-y (PMC10165757; doi:10.1186/s12906-023-03977-y)

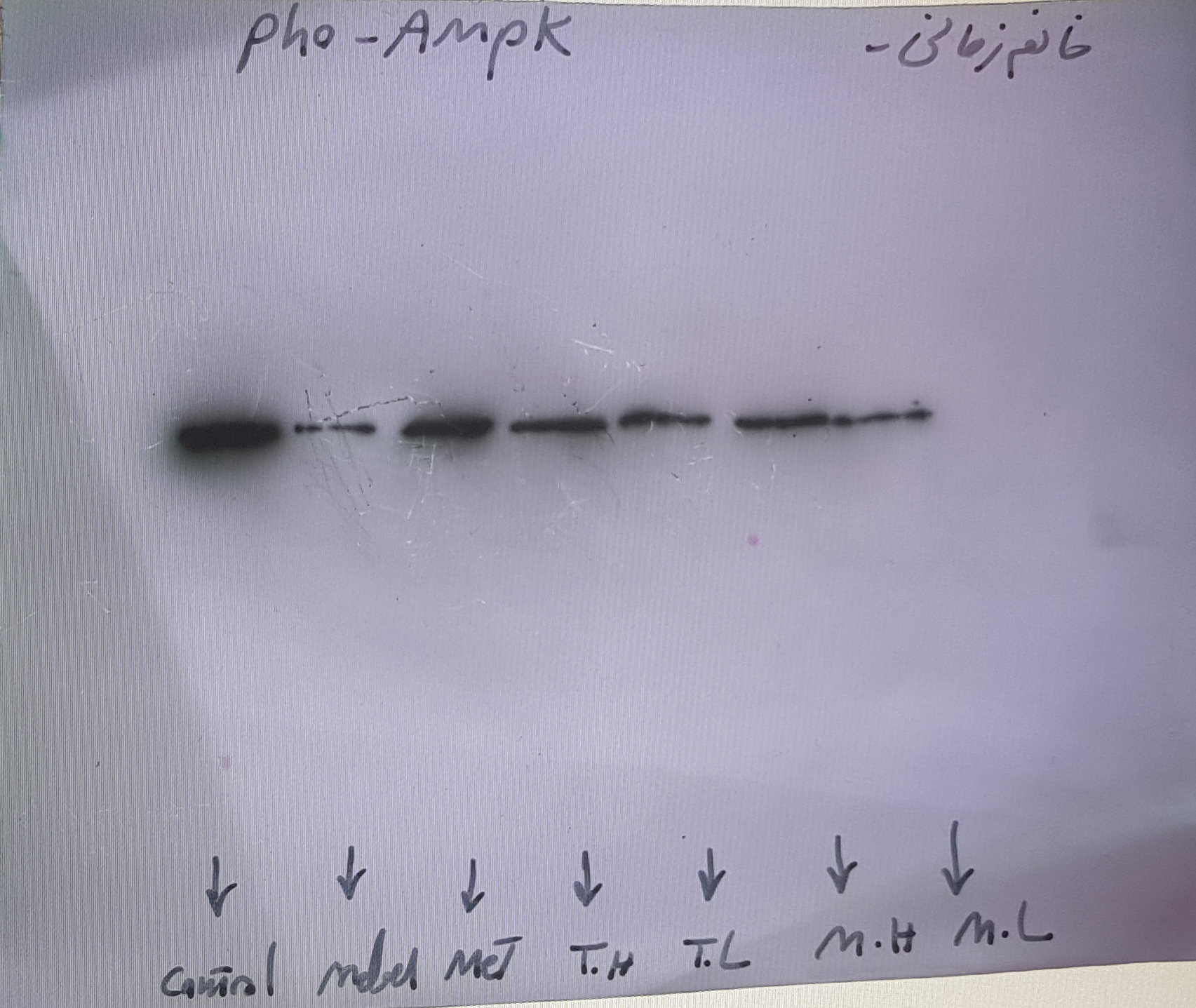

Supplement: Supplementary file 3 — Additional file 3 [file 12906_2023_3977_MOESM3_ESM.png]

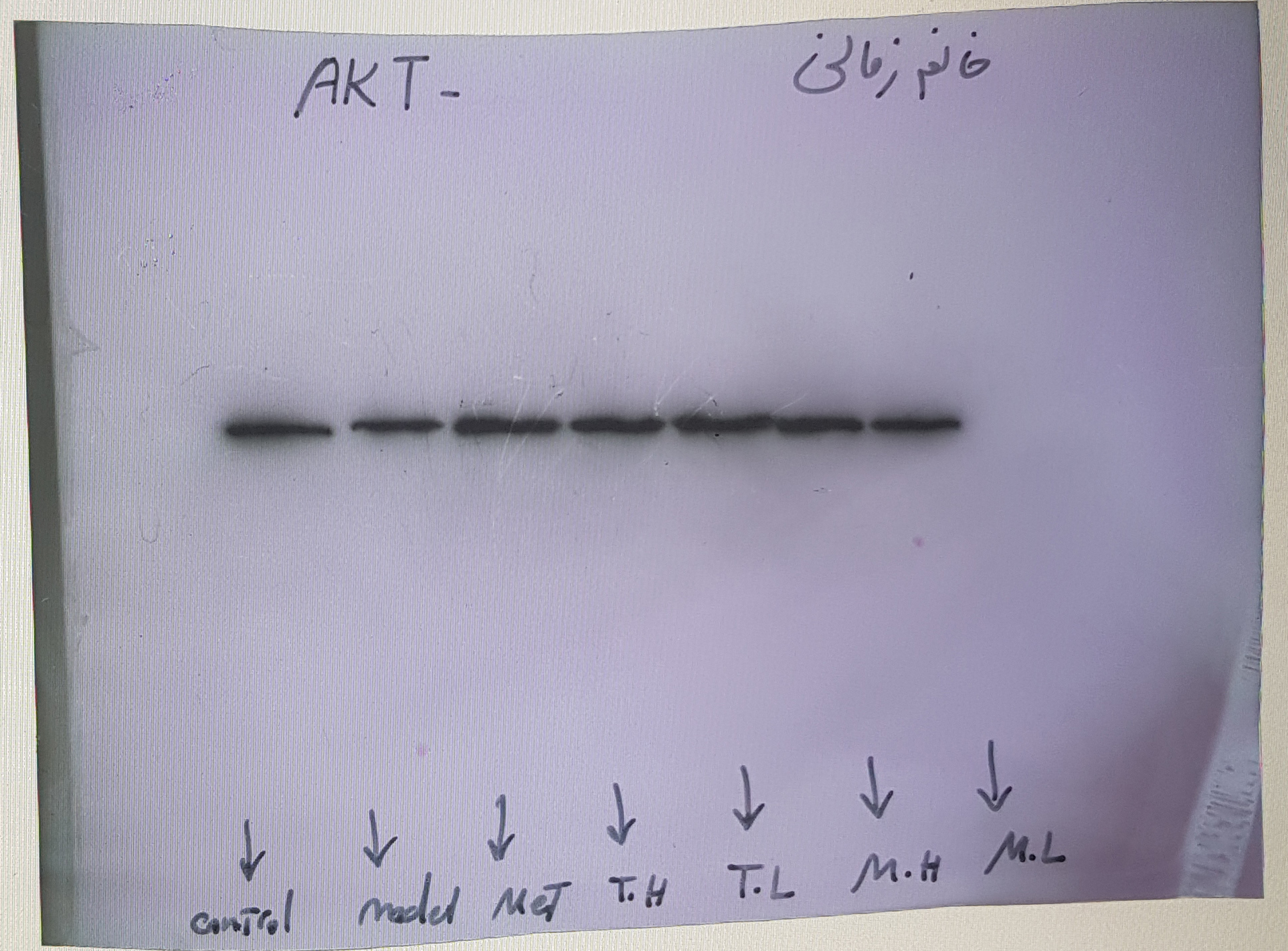

Supplement: Supplementary file 4 — Additional file 4 [file 12906_2023_3977_MOESM4_ESM.png]

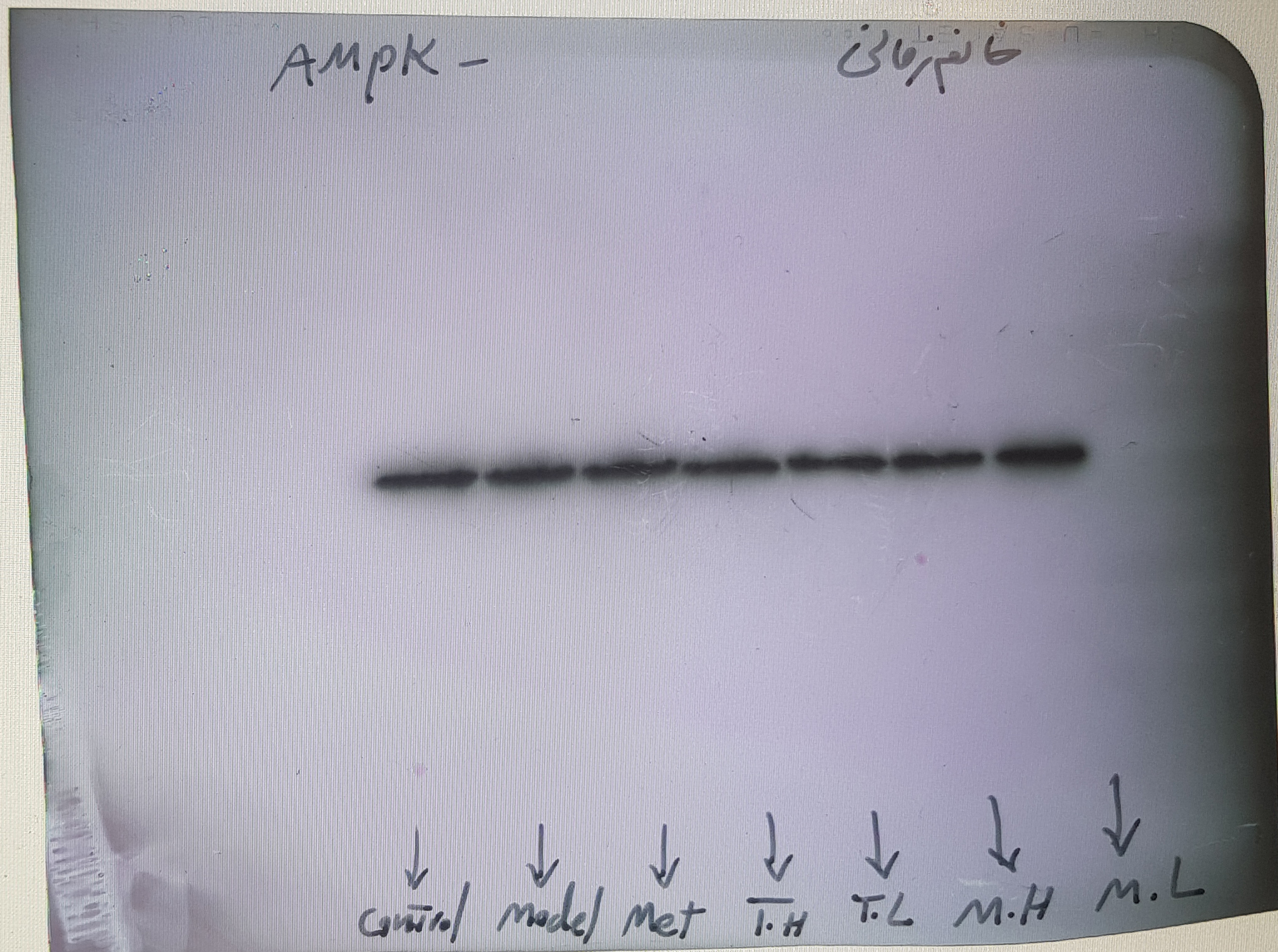

Supplement: Supplementary file 5 — Additional file 5 [file 12906_2023_3977_MOESM5_ESM.png]

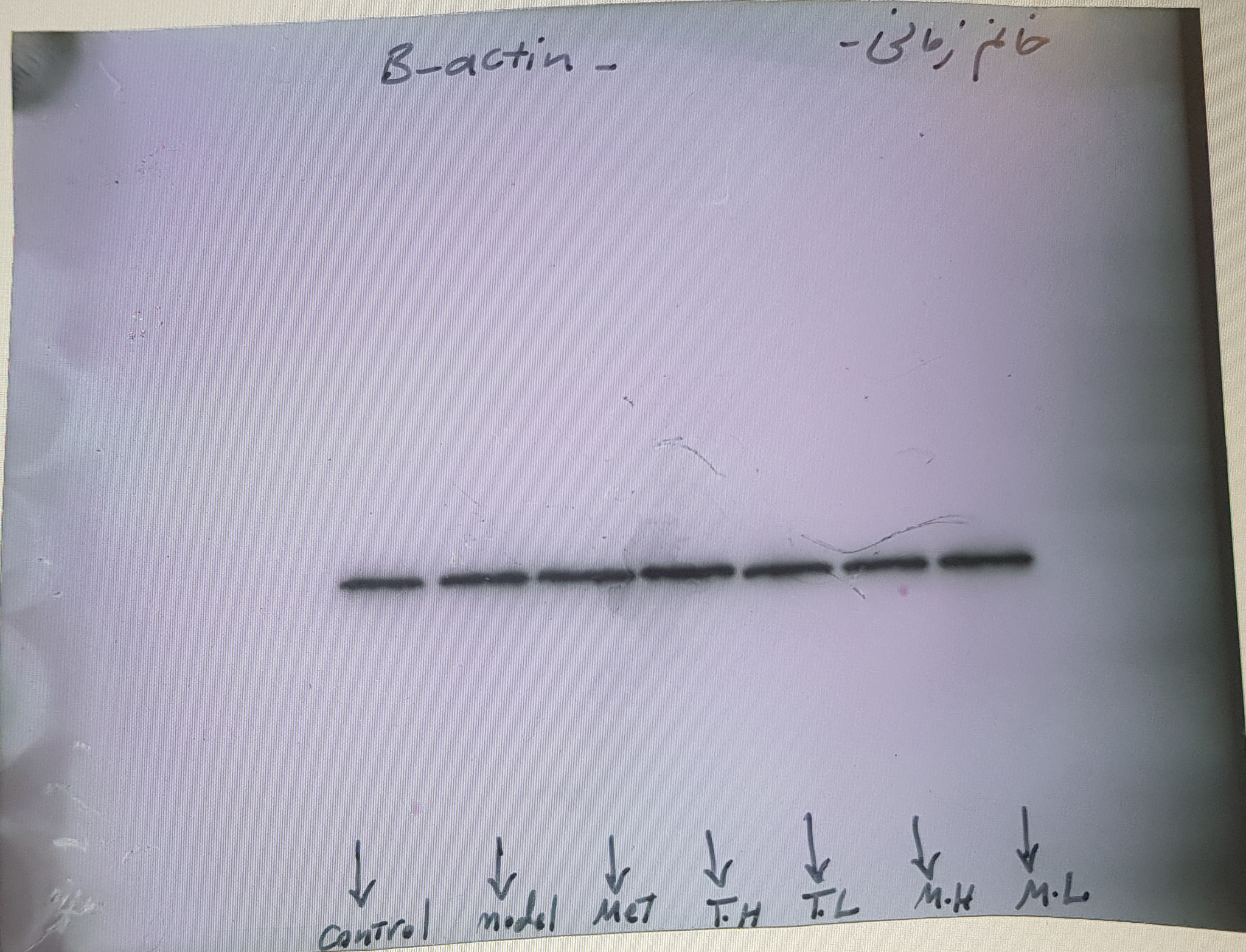

Supplement: Supplementary file 6 — Additional file 6 [file 12906_2023_3977_MOESM6_ESM.png]

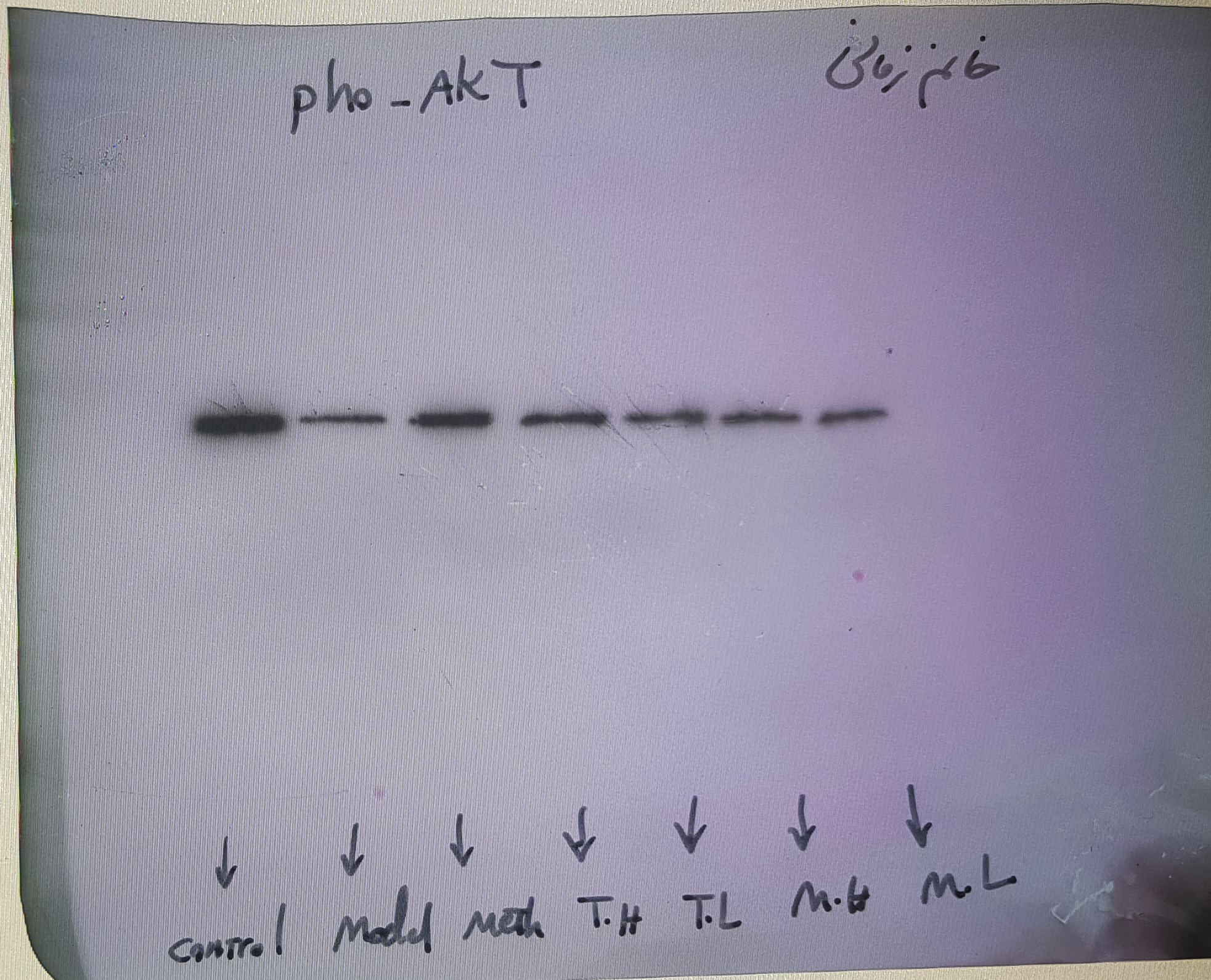

Supplement: Supplementary file 7 — Additional file 7 [file 12906_2023_3977_MOESM7_ESM.png]

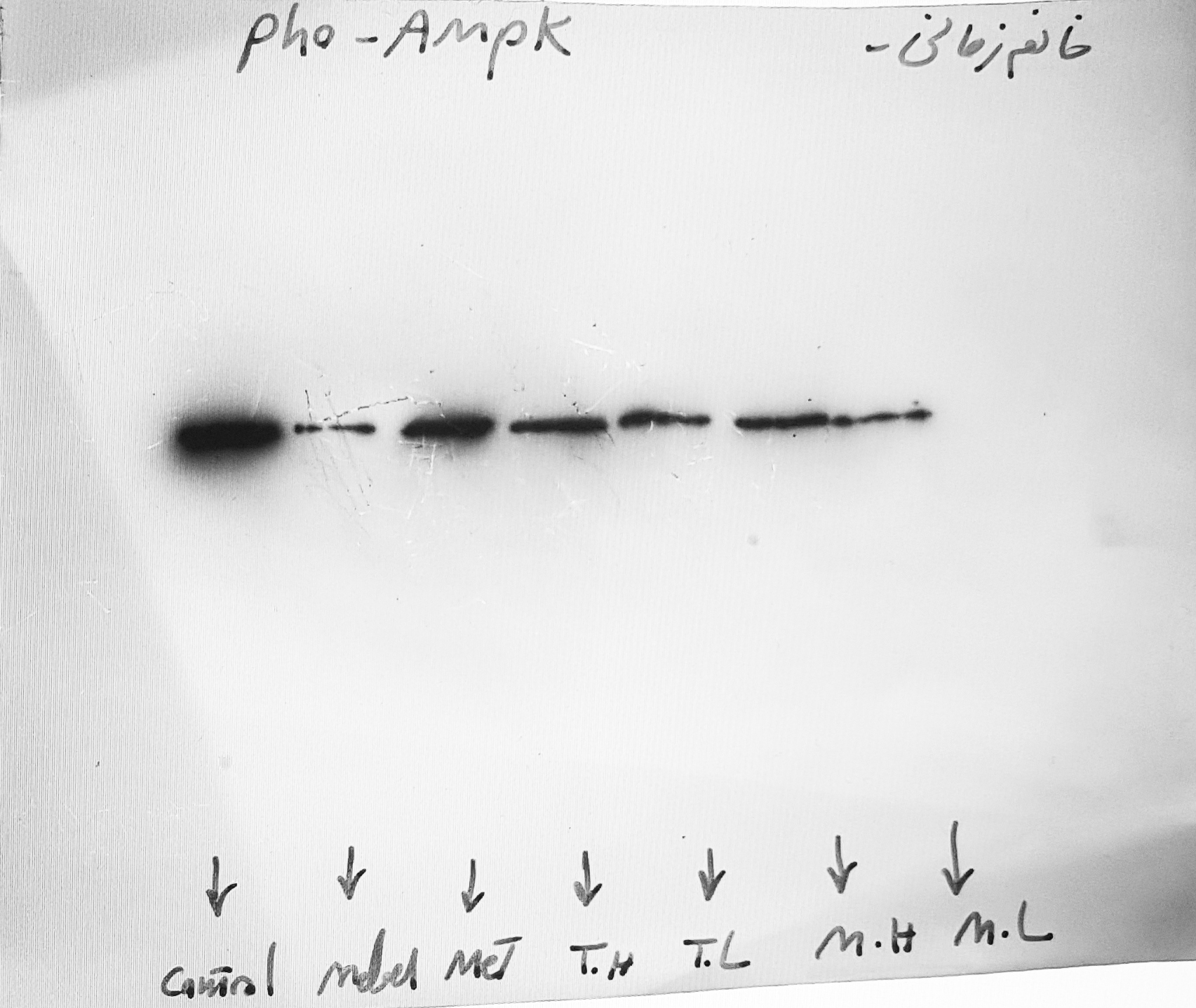

Supplement: Supplementary file 8 — Additional file 8 [file 12906_2023_3977_MOESM8_ESM.png]

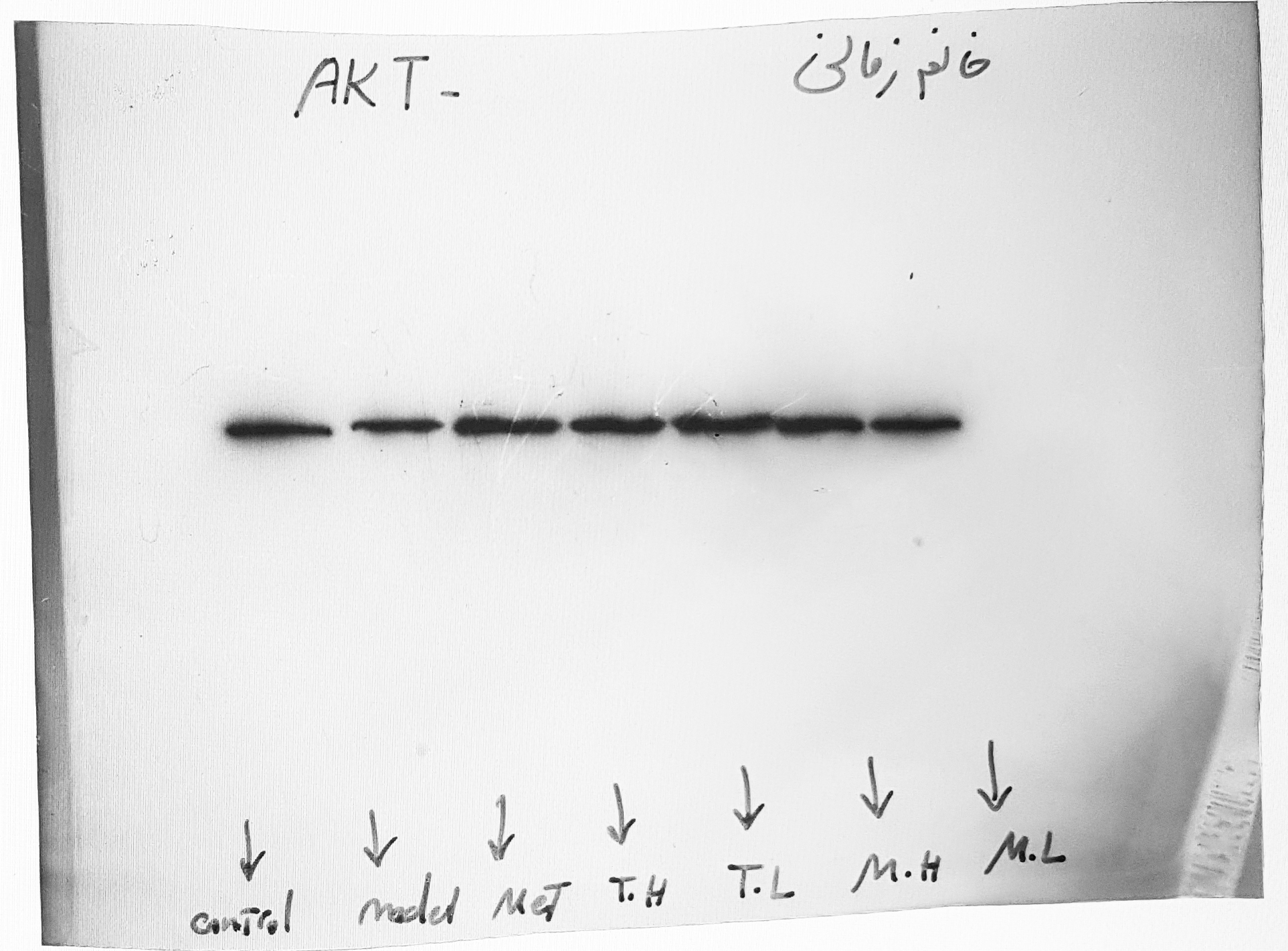

Supplement: Supplementary file 9 — Additional file 9 [file 12906_2023_3977_MOESM9_ESM.png]

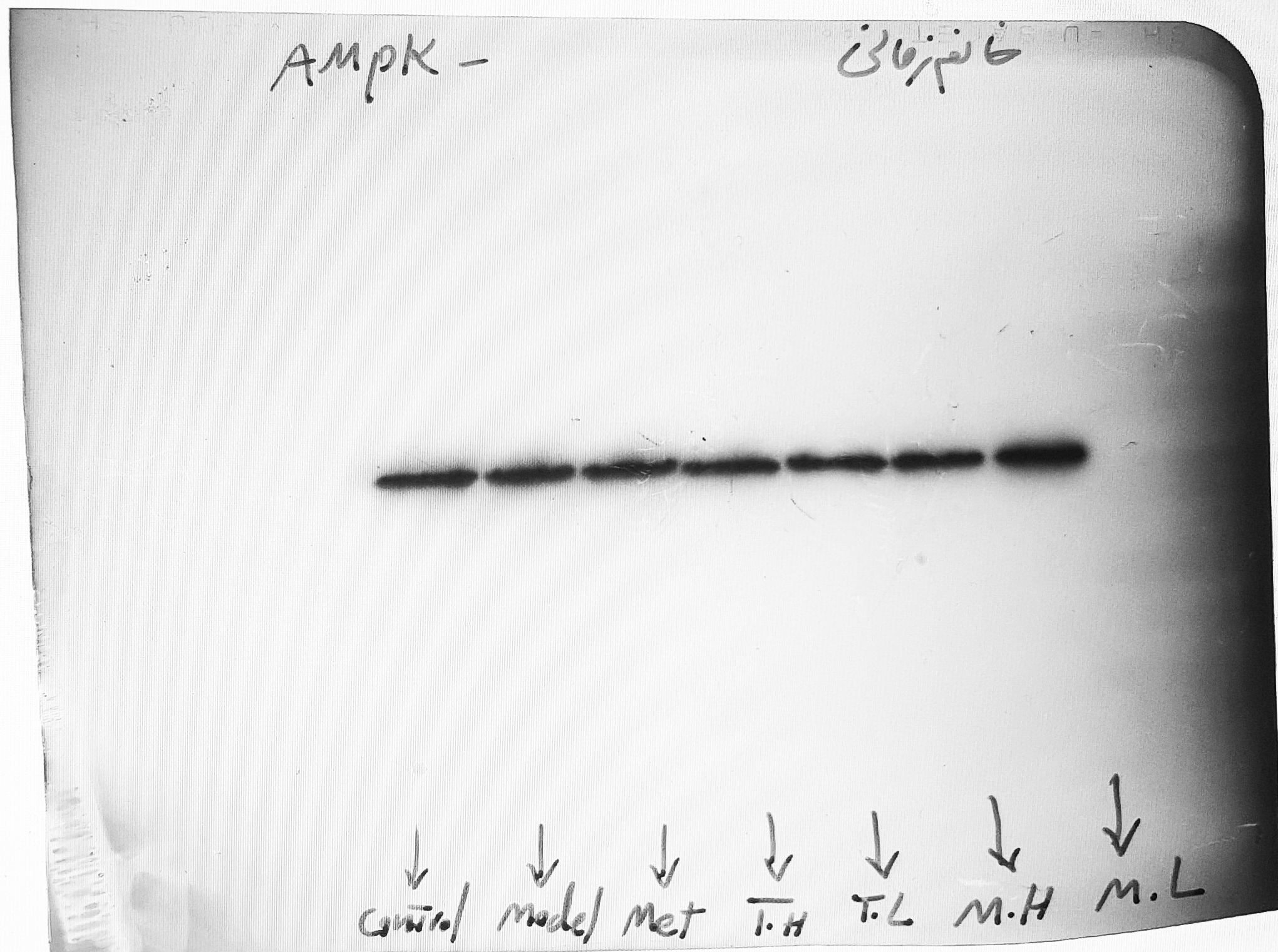

Supplement: Supplementary file 10 — Additional file 10 [file 12906_2023_3977_MOESM10_ESM.png]

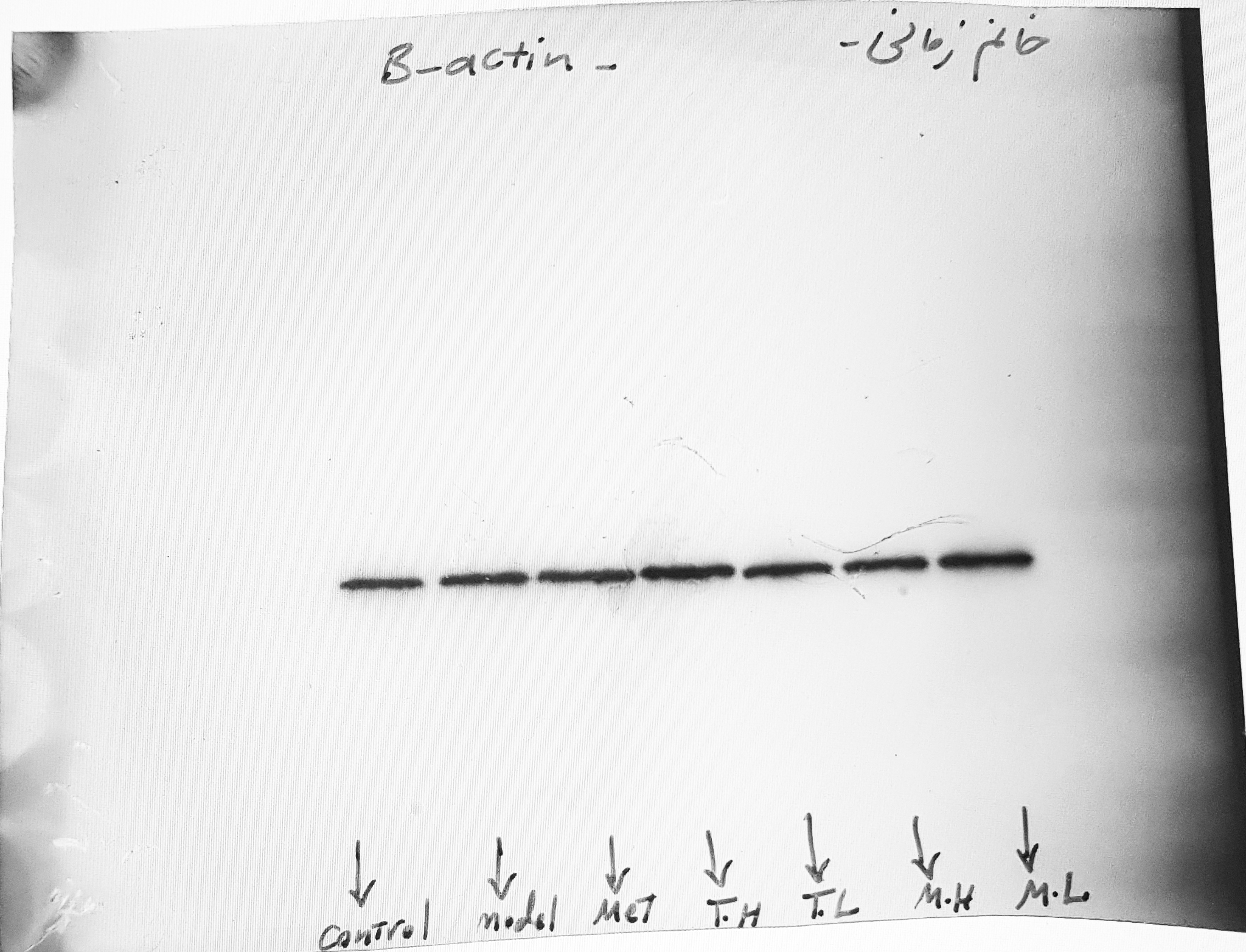

Supplement: Supplementary file 11 — Additional file 11 [file 12906_2023_3977_MOESM11_ESM.png]

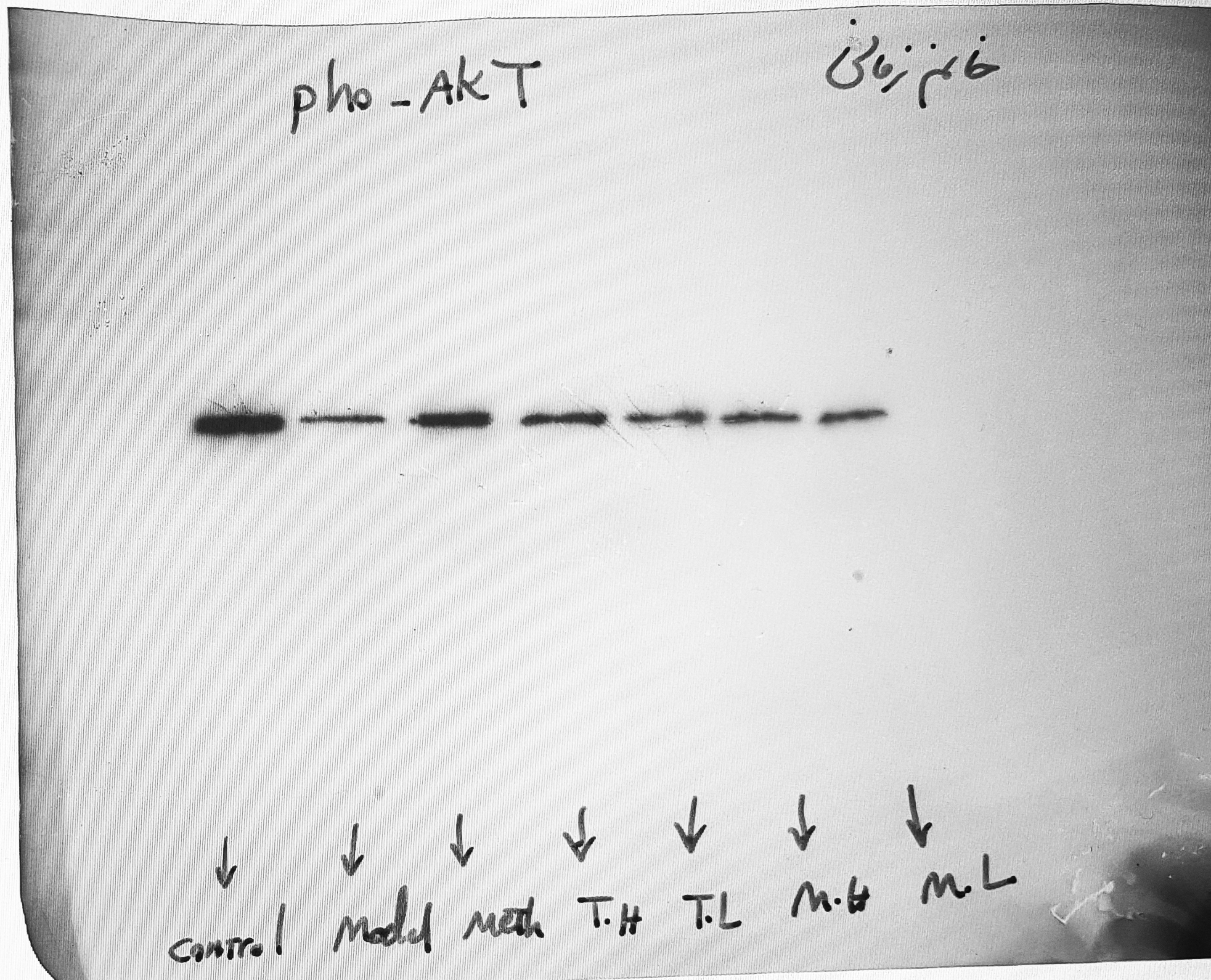

Supplement: Supplementary file 12 — Additional file 12 [file 12906_2023_3977_MOESM12_ESM.png]
